# Inhibitory effects of rat bone marrow-derived dendritic cells on naïve and alloantigen-specific CD4+ T cells: a comparison between dendritic cells generated with GM-CSF plus IL-4 and dendritic cells generated with GM-CSF plus IL-10

**DOI:** 10.1186/1756-0500-2-12

**Published:** 2009-01-23

**Authors:** George Tiurbe, Anja Matuschek, Ulrike Kämmerer, Manuela Schneider, Arnulf Thiede, Karin Ulrichs, Christoph Otto

**Affiliations:** 1 Experimental Transplantation Immunology Unit, Department of Surgery, University of Würzburg Hospital, Oberdürrbacher Str 6, D-97080 Würzburg, Germany; 2 Department of Obstetrics and Gynaecology, University of Würzburg Hospital, Josef-Schneider-Str 4, D-97080 Würzburg, Germany

## Abstract

**Background:**

Unlike mouse immature bone marrow (BM)-derived dendritic cells (DC), rat immature BMDC have not been thoroughly characterised *in vitro *for the mechanisms underlying their suppressive effect. To better characterise these mechanisms we therefore analysed the phenotypes and immune inhibitory properties of rat BMDC generated with GM-CSF plus IL-4 (= IL-4 DC) and with GM-CSF plus IL-10 (= IL-10 DC).

**Results:**

Both IL-4 DC and IL-10 DC exhibited lower surface expression of MHC class II and costimulatory molecules than mature splenic DC. They had a strong inhibitory effect on responsive T cells *in vitro *and despite their weak function as antigen-presenting cells they induced anergic T cells. However, only anergic T cells induced by IL-4 DC had a suppressive effect on responsive T cells. Induction of suppressive/tolerogenic T cells by IL-4 DC required direct contact between antigen-specific T cells and IL-4 DC. In addition, IL-4 DC and IL-10 DC prolonged allograft survival in an antigen-specific manner.

**Conclusion:**

A unique phenotype of immature BMDC was isolated from the cultures. The mechanisms underlying the suppressive effect may be caused by their inability to deliver adequate costimulatory signals for T-cell activation. In addition, IL-4 DC but not IL-10 DC induce anergic T cells with suppressive function. This indicates that IL-4 DC and IL-10 DC may differ in the quality of their costimulation although no differences in the surface expression of costimulatory molecules were found.

## Background

In recent years it has become clear that dendritic cells (DC) are not only potent inducers of adaptive immune responses, but also essential mediators in the induction and maintenance of T-cell tolerance [1]. The biological properties of DC depend on their phenotypically distinct states of development [2]. Their delaying effect on allograft rejection has been demonstrated in several rodent models [reviewed in [3]].

Mouse and human DC have both been studied thoroughly [reviewed in [4]]. Rat DC have been investigated particularly by groups interested in transplantation research [5-7]. They were not studied thoroughly, although established culture methods exist for the generation of bone marrow-derived rat DC (BMDC) [8,9]. The maturation of BMDC varies from species to species despite comparable culture conditions. In mice, for example, low doses of granulocyte macrophage colony stimulating factor (GM-CSF) combined with interleukin (IL)-4 induce the formation of mature BMDC [10], whereas in rats the same combination produces immature BMDC [11]. The effect of GM-CSF and IL-10 on the generation of rat BMDC is not clearly known.

In the present study we examined the ability of IL-4 DC and IL-10 DC to inhibit both the activation of naïve T cells and the restimulation of antigen-specific T cells *in vitro*. We also analysed their *in vivo *potential to prolong allograft survival.

## Methods

### Generation of rat BMDC

Femur and tibia bones of young (8–10 weeks) Lewis rats were extracted and disinfected in 70% ethanol. Both ends of the bones were cut and the bone marrow (BM) cells were flushed with 20 ml phosphate-buffered saline (PBS). The BM cells were cultured at a cell density of 5–8 × 10^5 ^cells/ml in culture dishes (Falcon, Becton Dickinson Biosciences). The RPMI 1640 culture medium [12] was supplemented with 5 ng/ml recombinant rat GM-CSF (R&D Systems, Heidelberg, Germany) and 5 ng/ml recombinant rat IL-4 (Miltenyi Biotech GmbH, Germany) or 5 ng/ml rat IL-10 (Miltenyi Biotech GmbH). On day 6, non-adherent cells and cells growing in clusters were collected.

### Cell isolation

Splenic DC (S-DC), naïve and antigen-specific T cells were isolated from Lewis rats as described previously [12]. Allopeptide P1-specific T cells were induced by immunization with P1 [12].

### Activation of naïve T cells and restimulation of antigen-specific T cells

Naïve T cells (10^5 ^cells/well) were incubated with 20 Gy irradiated IL-4 DC, IL-10 DC, or mature S-DC (10^4 ^cells/well) for 3 days at 37°C in a 5% humidified CO_2 _atmosphere. Allopeptide P1-specific T cells (10^5 ^cells/well) were incubated with irradiated (20 Gy) IL-4 DC, IL-10 DC, or S-DC (10^4 ^cells/well) loaded with P1 (1.25 μg/well) for 3 days at 37°C in a 5% humidified CO_2 _atmosphere. T-cell proliferation in 96-well, round-bottom plates was measured after ^3^H-thymidine (0.5 μCi/well) incubation for the last 6 h before harvesting. Radioactivity was determined as previously described [12]. Results (mean ± SD) were expressed in counts per minutes (cpm).

### Transwell experiments

Some of the assays were performed in 96-well transwell plates (Corning Life Sciences, The Netherlands). The upper compartment contained immature rat BMDC (1 × 10^4^), the lower compartment antigen-specific T cells (1 × 10^5^). After 3 days of culture, the transwells were removed and P1-loaded S-DC (10^4^/well) as well as antigen-specific T cells (1 × 10^5^/well) were added to the lower compartments. The cultures were then incubated for another 3 days and pulsed with 0.5 μCi/well [^3^H]-thymidine for the last 6 h of culture. The incorporation of [^3^H]-thymidine was measured as described [12].

### Heterotopic heart transplantation with and without antigen-loaded BMDC

The animal experiments were conducted in accordance with European, national and institutional animal care policies. Ten million BMDC were incubated with 20 μg P1 for 30 min in 500 μl PBS, washed 2 times with PBS and transferred intravenously via the penile vein into Lewis rats under full anesthesia with isoflurane 1 day before transplantation. Fully vascularised, heterotopic heart transplantation was performed, and graft survival was monitored as described [12].

## Results

### IL-4 DC and IL-10 DC exhibited an identical phenotype

**Figure 1:**
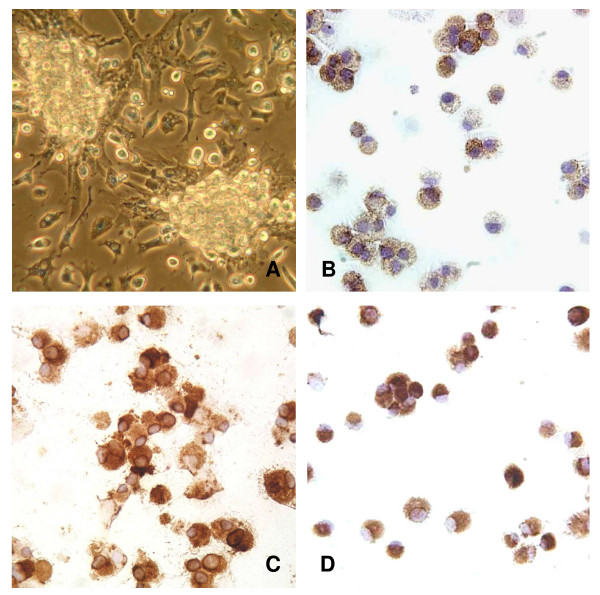
**Morphology and immunostaining of IL-4 DC and IL-10 DC**. Rat BMDC were isolated from cell clusters on day 6 of culture (A). Cells prepared on cytospin slides stained positive for monoclonal antibodies Ox62 (rat DC marker) (B), Ox6 (MHC class II) (C), and CD68 (D). Shown are representative IL-4 DC results, which are similar to those for IL-10 DC. Magnification: ×200 (A, C, D) and ×400 (B).

**Figure 2:**
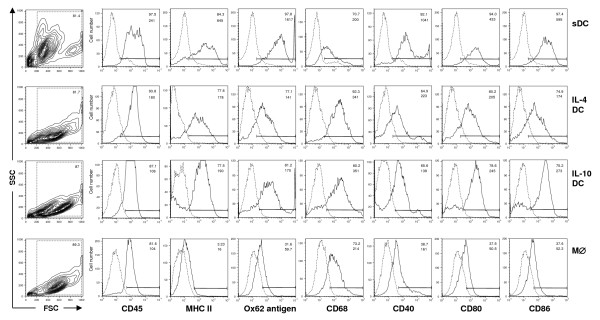
**IL-4 DC and IL-10 DC exhibit no obvious differences in their phenotype**. IL-4 DC and IL-10 DC and mature splenic DC (sDC) coexpressed Ox62 and CD68, whereas macrophages generated in the presence of M-CSF (5 μg/ml) were only positive for CD68. Broken lines indicate background staining obtained using an irrelevant isotype control. The first number represents the percentage of cells staining positive for the indicated marker and the second number represents the mean fluorescence intensity. The results shown are representative for 4 independent flow cytometric analyses. The antibodies were purchased from Serotec, Ltd, Oxford, United Kingdom, with the exception of HM40-3 and 24F (BD Biosciences, Heidelberg, Germany).

On approximately day 3 of culture low adherent cell clusters were obvious and both the number and size of the clusters increased during culture (Fig. 1A). The BMDC isolated from these clusters on day 6 were positive for Ox62 (a marker for rat DC), Ox6 (anti-MHC class II), and the macrophage marker ED1 (CD68) (Fig. 1B–D). More than 77% of the cells isolated from the clusters were double positive for Ox62 and Ox6 without further purification. The flow cytometric analysis revealed that IL-4 DC and IL-10 DC expressed MHC II and the costimulatory molecules CD40, CD80 and CD86 at nearly identical levels on their surface (Fig. 2).

### IL-4 DC and IL-10 DC neither activated naïve T cells nor restimulated antigen-specific T cells

**Figure 3:**
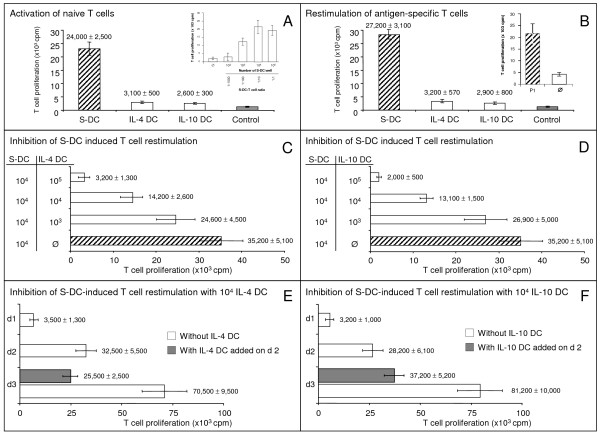
**IL-4 DC and IL-10 DC inhibit the proliferation of responsive T cells**. IL-4 DC and IL-10 DC were unable to activate either naïve T cells in the mixed leukocytes culture (A) or antigen-specific T cells in the T-cell proliferation assay (B). The effect of mature S-DC on the proliferation of naïve T cells is shown in the inset of Fig. A. The antigen-specific restimulation of P1-specific T cells by P1-loaded S-DC (P1) is shown in B (inset). Different numbers of IL-4 DC and IL-10 DC influenced the S-DC-induced proliferation of antigen-specific T cells (C-D). The addition of P1-loaded IL-4 DC and IL-10 DC to the proliferation assay on day 2 of the 3-day culture prevented the marked increase in T-cell proliferation that had been induced by P1-loaded S-DC between days 2 and 3 (E-F). The results (mean ± standard deviation) shown are representative for 3 different experiments. Control: Naïve T cells (A) and antigen-specific T cells (B) cultured alone; ∅: P1-unloaded S-DC.

Naïve T cells did not proliferate in the presence of IL-4 DC and IL-10 DC, whereas mature S-DC induced a strong T-cell proliferation (Fig. 3A). IL-4 DC and IL-10 DC loaded with the allogeneic peptide P1 [12] were not able to restimulate P1-specific T cells (Fig. 3B). Different numbers (10^3^, 10^4 ^and 10^5^) of P1-loaded IL-4 DC or IL-10 DC suppressed the proliferation of antigen-specific T cells induced by P1-pulsed S-DC (Fig. 3C, D). For both BMDC types, IL-12-specific mRNA was not detectable by RT-PCR (Additional File 1, Table 1). This may indicate that they revealed properties of immature DC.

**Table 1:** Primers and PCR conditions.

**Primer**	**Sequence (5'- > 3')**	**PCR product**	**Annealing temperature**
GAPDH	For: GGT CGG TGT GAA CGG ATT TG Rev: GTG AGC CCC AGC CTT CTC CAT	319 bp	62°C

MHC II	For: CAG GAT CTG GAA GGT CCA Rev: AGC TGT GGT TGT GCT GA	517 bp	55°C

CD40	For: CGC TAT GGG GCT GCT TGT TGA CAG; Rev: GAC GGT ATC AGT GGT CTC AGT GGC	401 bp	58°C

CD80	For: TGG TGA AAC ACC TGA CCA Rev: GTT TCT CTG CTT GCC TCA	517 bp	50°C

CD86	For: TGG GAA ACA GAG CTC TCA Rev: AGG TTG ATC GAC TCG TCA	518 bp	53°C

Primers specific for rat IL-12p35 was purchased from Invitrogen/Biosource. Total RNA was isolated from 10^6 ^cells with 1 ml of the ready-to-use reagent Trizol Reagent (Invitrogen GmbH, Germany) according to the manufacturer's recommendations. Reverse transcription of 5 μl RNA was performed using the GeneAmp RNA-PCR-Kit (Applied Biosystems GmbH, Germany). Five μl cDNA was amplified using Gold AmpliTaq DNA-Polymerase (0.05 U/μl) and the specific primers (5 μmol/L each) mixed in nuclease free water (Promega GmbH, Germany) to an end volume of 50 μl per sample.

In the BMDC-uninfluenced T-cell proliferation assay, the strongest increase in proliferation of antigen-specific T cells occurred between days 2 and 3 of the 3-day culture. The addition of P1-pulsed IL-4 DC or IL-10 DC to the cultures on day 2 halted this strong increase in T-cell proliferation within 24 h (Fig. 3E, F).

### IL-4 DC-T and IL-10 DC-T showed anergic properties

**Figure 4:**
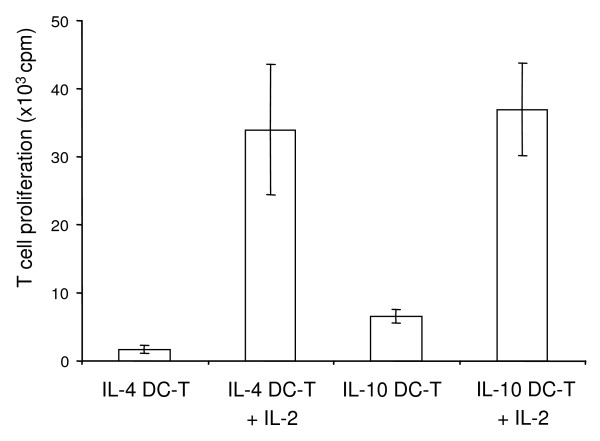
**IL-4 DC-T and IL-10 DC-T do not proliferate and demonstrate anergy**. IL-4 DC-T and IL-10 DC-T, purified and transferred to second cultures, did not proliferate in response to subsequent stimulation with P1-loaded splenic DC (S-DC), but required the addition of IL-2 (B). In contrast, antigen-specific T cells not precultured with IL-4 DC or IL-10 DC had proliferation rates of 35,200 ± 5,100 cpm (not shown). The results (mean ± standard deviation) are representative for 3 different experiments.

P1-specific T cells were named IL-4 DC-T when incubated with IL-4 DC and IL-10 DC-T when incubated with IL-10 DC. Following purification, the DC-T were transferred to 10^4 ^P1-pulsed mature S-DC and their proliferation rate after 72 h was low (Fig. 4). The anergic-like effect of IL-4 DC-T and IL-10 DC-T could be negated by adding exogenous IL-2 (27 ng/ml) to the cultures (Fig. 4).

### IL-4 DC-T had an inhibitory effect on antigen-specific T cells

**Figure 5:**
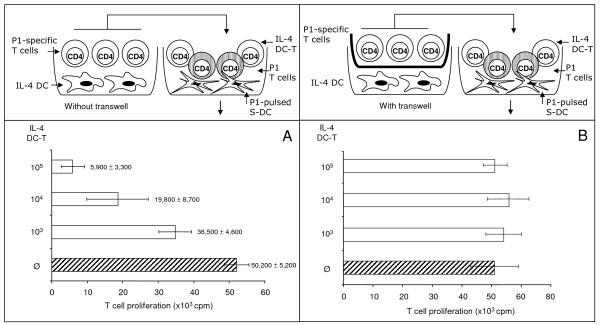
**IL-4 DC-T inhibit the proliferation of antigen-specific T cells in a dose-dependent manner**. Different numbers of IL-4 DC-T which were in contact with IL-4 DC during the first culture (without transwell) were added to the proliferation assay containing freshly isolated P1-specific T cells and P1-loaded S-DC. The IL-4 DC-T inhibited their restimulation in a cell number dependent manner (A). Antigen-specific T cells which were not in contact with IL-4 DC during the first culture (transwell experiment) had no inhibitory effect (B). The results (mean ± standard deviation) are representative for 3 different experiments.

Different numbers (10^3^, 10^4 ^and 10^5^) of IL-4 DC-T were added to assays containing P1-loaded S-DC and freshly isolated antigen-specific T cells. Measurement of proliferation revealed that IL-4 DC-T had a dose-dependent inhibitory effect on T-cell proliferation (Fig. 5A). Antigen-specific T cells cultured with IL-4 DC-T in transwell plates did not reduce the control T-cell proliferation (Fig. 5B). Antigen-specific T cells must have direct contact with IL-4 DC in precultures to have an inhibitory effect on T-cell proliferation in second cultures. The IL-10 DC-T, in contrast, had no influence on the proliferation of antigen-specific T cells (not shown).

### P1-loaded IL-4 DC and IL-10 DC prolonged antigen-specific cardiac allograft survival

**Figure 6:**
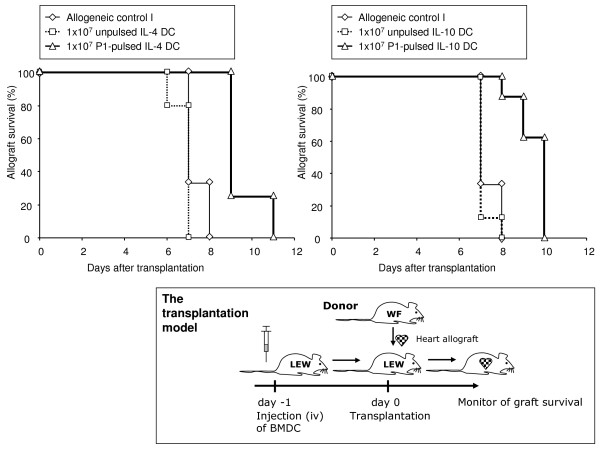
**The *in vivo *effect of P1-loaded IL-4 DC and IL-10 DC**. P1-loaded IL-4 DC and IL-10 DC prolonged the survival of allogeneic hearts grafts from Wistar-Furth in Lewis recipients. In contrast, unpulsed IL-4 DC and IL-10 DC did not influence allograft survival. The allogeneic control group I demonstrates the natural rejection of Wistar-Furth allografts by Lewis recipients (see also Table 2).

Ten million of P1-loaded BMDC administered intravenously to each Lewis rat 1 day before they received heart allografts let them survive 2 days longer than those animals transfused with unpulsed BMDC, which had no effect on allograft survival (Table 2 and Fig. 6). The survival of allografts from third party donors (Brown-Norway rats) was not affected by the transfer of P1-pulsed IL-4 DC and IL-10 DC (Table 2).

**Table 2:** The effects of allopeptide pulsed and unpulsed IL-4 DC and IL-10 DC *in vivo*.

**Group**	**Origin of DC**	**Donor of heart allografts**	**Survival time (d) of heart allografts in LEW recipients**	**MST ± SD (d)^1)^**	**Median (d)**	**n**
1 Syngeneic Ctr	---^2)^	LEW	> 50(×3)	> 50	50	3

2 Syngeneic Ctr + P1	---	LEW	> 50(×3)	> 50	50	3

3 Allogeneic Ctr I	---	WF	7(×4), 8(×2)	7.3 ± 0.5	7.0	6

4 Allogeneic Ctr I + P1^3)^	---	WF	5(×4), 6(×2)	5.3 ± 0.5	5.0	6

5 1 × 10^7 ^IL-4 DC	LEW ^4)^	WF	6, 7(×4)	6.8 ± 0,5	7.0	5

6 1 × 10^7 ^IL-4 DC + P1	LEW	WF	9(×3), 11	9.5 ± 1.0	9.0	4

7 1 × 10^7 ^IL-10 DC	LEW	WF	7(×7), 8	7.1 ± 0.4	7.0	8

8 1 × 10^7 ^IL-10 DC + P1	LEW	WF	8, 9(×2), 10(×5)	9.5 ± 0.8	10	8

9 Allogeneic Ctr II	---	BN	7(×3)	7.0	7.0	3

10 Allogeneic Ctr II + P1	---	BN	7(×3),8	7.3 ± 0.5	7.0	4

11 1 × 10^7 ^IL-4 DC	LEW	BN	7(×3)	7.0	7.0	3

12 1 × 10^7 ^IL-4 DC + P1	LEW	BN	7(×2), 8	7.3 ± 0.6	7.0	3

13 1 × 10^7 ^IL-10 DC	LEW	BN	7(×3)	7.0	7.0	3

14 1 × 10^7 ^IL-10 DC + P1	LEW	BN	7(×3)	7.0	7.0	3

^1) ^Mean survival time ± standard deviation in days (d).

^2) ^No DC treatment performed.

^3) ^Animals were immunised with P1 7 days prior to transplantation.

^4) ^LEW: Lewis rats, WF: Wistar Furth rats, BN: Brown Norway rats.

## Discussion

In the present study, we compared the effects of 2 types of rat BMDC on the proliferation of naïve and antigen-specific T cells *in vitro *and on the survival of allogeneic heart allografts. Both DC types displayed lower surface expression of MHC class II and costimulatory molecules compared to mature splenic DC. IL-4 DC and IL-10 DC had a strong inhibitory effect on responsive T cells. This suppressive effect was detectable within 24 h after the BMDC were added to cultures with antigen-specific T cells and mature splenic DC (Fig. 3E, F). To our knowledge this is the first description of the time course BMDC needed to suppress T-cell proliferation. We found that antigen-specific T cells became anergic after incubation with IL-4 DC and IL-10 DC. The same effect has been described for human IL-10 modified DC on CD4+ T cells [13]. Anergic T cells isolated from cultures with P1-loaded IL-4 DC suppressed the DC-mediated activation of responsive T cells in a cell count-dependent manner. They share this suppressive effect with regulatory T cells [14]. We hypothesise that IL-4 DC and IL-10 DC may differ in the quality of their costimulation, with IL-4 DC inducing suppressive IL-4 DC-T whereas IL-10 DC do not. It should be emphasised, however, that we found no differences in the surface expression of costimulatory molecules between the two BMDC types.

The results of transwell experiments showed that the contact between IL-4 DC and the antigen-specific T cells is a prerequisite for inducing suppressive IL-4 DC-T. However, we made no attempt in this study to determine whether the anergic IL-4 DC-T mediate their suppressive effect via cell-cell contact or by soluble factors. Vendetti et al., for example, reported that inhibition mediated by anergic murine T cells is dependent on cell-cell contact [15]. They also described an inhibitory effect of anergic T cells on the antigen-presenting function of mature DC.

IL-4 DC and IL-10 DC, loaded with allopeptide P1, prolonged allograft survival (Fig. 6). In addition, survival time can be improved by increasing the number of transferred cells. The application of 30 million P1-pulsed IL-10 DC, for example, prolonged survival time to a median of 10.6 ± 0.8 days (not shown) from the 9.5 ± 0.8 days achieved with 10 million P1-pulsed IL-10 DC. Allograft survival was prolonged only when the BMDC were pulsed with the immunodominant allogeneic peptide P1 involved in allograft rejection [5]. Our results accord with those of Chowdhury et al. [16], who showed that the presentation of allogeneic peptides by tolerogenic thymic rat DC greatly prolongs allograft survival. Compared to the results of Chowdhury et al., our 2–3 day prolongation of allograft survival may seem meager, but considering the strength of the allogeneic immune response induced by alloreactive T cells and the fact that no immunosuppressive drugs were used, our findings appear very promising.

## Conclusion

The data suggest that rat IL-4 DC and IL-10 DC have suppressive/regulatory properties comparable to those described for immature mice BMDC. They demonstrate a strong inhibitory effect on responsive T cells, probably the consequence of a reduced surface expression of costimulatory molecules paired with the inability to deliver adequate costimulatory signals to T cells. IL-4 DC and IL-10 DC are identical in phenotype and in some of their effects, but they are different in their capacity to induce suppressive T cells. IL-4 DC induce T cells with suppressive/regulatory function whereas IL-10 DC do not. This may indicate that IL-4 DC and IL-10 DC differ in the quality of their costimulation. Further studies are necessary to test this hypothesis.

## Competing interests

The authors declare that they have no competing interests.

## Authors' contributions

GCT designed the study, set up the experiments, collected the data, co-drafted the manuscript and provided images and figures. AM set up the flow cytometric experiments, participated in data collection, analysed and interpreted the results, and provided images and figures. UK revised the article for scientific content. MS performed the transplantation experiments and carried out the histological analysis. AT participated in editorial support and research funding. KU assisted in the study design, experimental concept, and data interpretation. CO drafted the manuscript, designed the study, analysed and interpreted the results and provided images and figures. All authors read and approved the final manuscript.

## Supplementary Material

Additional file 1 **IL-4 DC and IL-10 DC do not express IL-12.** Shown are the results of reverse transcriptase-polymerase chain reaction (RT-PCR). The used primers are listed in Table 1. Click here for file
