# Supplementary figures and images for: Inhibitory effects of rat bone marrow-derived dendritic cells on naïve and alloantigen-specific CD4+ T cells: a comparison between dendritic cells generated with GM-CSF plus IL-4 and dendritic cells generated with GM-CSF plus IL-10
Source: BMC Res Notes. 2009 Jan 23;2:12. doi: 10.1186/1756-0500-2-12 (PMC2639598; doi:10.1186/1756-0500-2-12)

IL-4 DC    IL-10 DC    S-DC

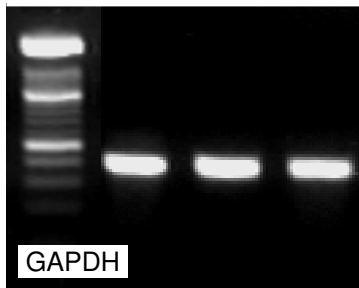

IL-4 DC    IL-10 DC    S-DC

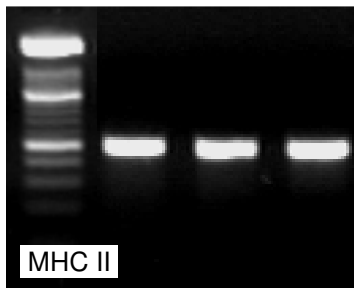

IL-4 DC    IL-10 DC    S-DC

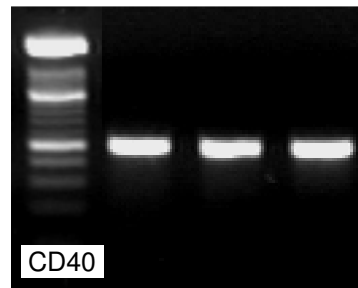

IL-4 DC    IL-10 DC    S-DC

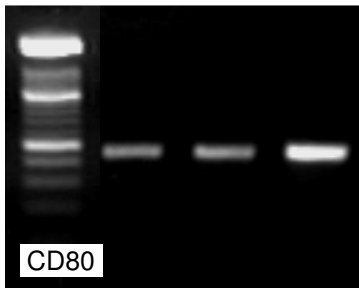

IL-4 DC    IL-10 DC    S-DC

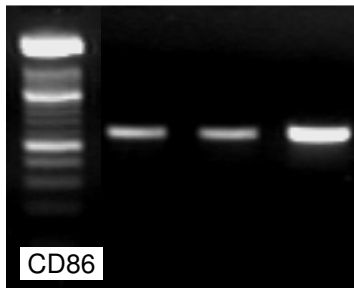

IL-4 DC    IL-10 DC    S-DC

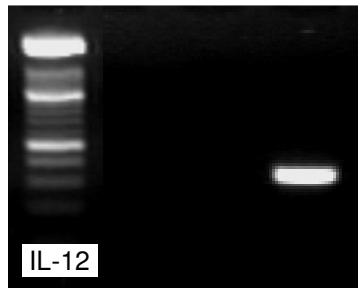

Supplement: Additional file 1 — IL-4 DC and IL-10 DC do not express IL-12. Shown are the results of reverse transcriptase-polymerase chain reaction (RT-PCR). The used primers are listed in Table 1. [file 1756-0500-2-12-S1.pdf]
